# Progress in Medicine

**Published:** 1895-08-24

**Authors:** 


### Progress in Medicine.

### GRAVES' DISEASE.

On the physiology and pathology of the thyroid gland much light has been shed during the past ten years; and more recently the discovery that myxœdema, cretinism, and cachexia strumipriva are produced by absence or atrophy of the thyroid gland, and may be favourably modified, and generally cured, by administering the gland by the mouth, has shown that the thyroid gland either destroys some substance which otherwise accumulates in the blood and is deposited in the tissues, or that it secretes something which is necessary to the economy, and without which cretinism and myxœdema will arise. But it is now very generally known that often the result of taking more than moderate doses of the thyroid gland, or its extract, is that a symptomatic complex is produced, indistinguishable from true Graves' disease; and the view that while myxœdema is due to deficient activity of the thyroid gland, Graves' disease, or exophthalmic goitre, is due to excessive functional activity of the gland is gaining ground, and has already been made the basis of a new treatment of the disease under discussion. It will be useful to examine the data bearing on these points which recent literature has furnished.

**Physiology of the Thyroid Gland.**—Gley[1] has drawn attention to the structural changes determined in the accessory thyroid glandules by extirpation of the thyroid gland proper in rabbits. Under normal condition the glandules are of a homogeneous structure. On the contrary, within six days, and still more distinctly after fifteen or eighteen days have elapsed after extirpation of the thyroid gland, sections under a feeble magnifying power, show irregularly disseminated areas formed by cross bars of epithelial cells of smaller size than normal ones, with deeply stained protoplasm and a nucleus which stains more readily than the nuclei of normal elements. Not only is this trabecular structure well marked, but, what is of still greater importance, the epithelial cell elements have acquired characters which the cells of normal glands never possess. The hypertrophy of these organs is, therefore, due, if not entirely, at any rate in a great measure, to increase in the number of their epithelial cells. Moreover, in some cases phenomena of cellular division have been observed; but so far we have not discovered any vesicles with colloid contents. Gley thinks that these cellular modifications are due to functional development of the glandules, as a consequence of the thyroidectomy, and that in this manner such ill-effects as invariably result from total extirpation in rabbits of both glands and glandules are obviated.

We now turn to Notkine's[2] investigations into the nature of the substance in the thyroid which produces the effects following ingestion of the gland. Assuming that cachexia strumipriva is the result of intoxication by one or more toxic products, which accumulate in the organism of a thryroidectomised animal, he regarded this cachexia in the light of a true auto-intoxication, inasmuch as it is observed even in animals subjected after the operation to absolute deprivation of food, although in this case it is much less marked than if they are fed. The thyroid gland, then, secretes a substance which is capable of decomposing or neutralising the toxic products of the intra-organic exchanges, products which are the cause of the phenomena of cachexia strumipriva in thyroidectomised animals. Now a large number of experiments on animals, as well as clinical observations on a case of myxœdema led Notkine to the conclusion that the toxic principle which determines the cachexia strumipriva is to be found in the thyroid gland itself, and he has succeeded in extracting from the thyroid gland of various animals a peculiar albuminoid substance, constituting the greater part of the colloid mass of the thyroid gland, and to which he has given the name of thyro-proteid.

From experiments with this thyro-proteid he arrived at the following conclusions: (1) That thyro-proteid belongs chemically to the albuminoid group. (2) That chemically pure thyro-proteid is toxic to animals, and determines symptoms analogous to those of cachexia strumipriva, and is very slowly decomposed in the organism, and very slowly eliminated, and has, therefore, a cumulative effect. (3) To an animal deprived of the principal part of the thyroid gland, thyro-proteid is toxic, even in doses which normal animals can bear without difficulty; but if after partial thyroidectomy the injection of thyro-proteid is delayed until the resected gland has had time to become hypertrophied, the animal operated upon supports the thyro-proteid just as well as a normal animal. (4) The effect of thyro-proteid is first stimulating, then paralysing; it is probable that it acts on the central nervous system. The contractions of the heart appear to be slackened; the general nutrition is affected, emaciation occurring in all cases in which the action of the thyro-proteid is slow. Notkine believes that the colloid substance in diffuse colloid goitres represents the anatomical equivalent, as it were, of the thyro-proteid, and that the latter is not a product of secretion of the thyroid gland, but the waste material of intra-organic changes. The thyro-proteid, in his opinion, constitutes the poison which accumulates in the organism after the operation of thyroidectomy, and determines myxœdemic phenomena, but this poison is destroyed or neutralised by the genuine product of the secretion of the thyroid gland, which contains a special ferment. The physiological rôle of the thyroid gland, then, according to Notkine's views, is to rid the organism of the thyro-proteid existing in the blood; to store up this toxic substance in the alveoles of the gland, where it is neutralised and rendered harmless, after which it is again poured into the circulation. He has observed that Graves' disease, which "probably is the result of intoxication by thyroid enzyme, produced in excess," appears to be influenced in an extremely favourable manner by the administration of thyro-proteid in small doses. Other facts bearing on the functions of the thyroid and its relation to Graves' diseases have been adduced by Edmunds,[3] who has demonstrated that in exophthalmic goitre there is as a rule little colloid substance, but solid cylindrical processes of cells compose the main structure; similar tissue is met with in ordinary goitres. He takes the view that the general symptoms in Graves' disease were due to an excessive secretion, not of colloid, but some other substance, and the passage of this into the circulation. The particular tissue that he found resembled that of the accessory thyroids in animals and also in the human subject. He drew attention to the fact that the secretion of the common goitre does not cause exophthalmous. He had given a dog sixteen sheep's thyroids a day without producing any result; but the protrusion of the eye could be produced by cocaine internally or externally applied to the conjunctiva, while division of the cervical sympathetic caused the eye to recede.

Thus it would appear that Graves' disease is associated with excessive activity of certain epithelial tissues in the thyroid gland, and that these secrete a substance which produces the peculiar phenomena of the disease, but which ordinarily and in normal amount neutralise and destroy the colloid substance, which otherwise would accumulate in the system and give rise to myxœdema.

[1] Med. Week., Mar. 27, 1895. [2] Med. Week, May 3, 1895. [3] British Med. Jour., May 25, 1895.

*(To be continued.)*

